# Encapsulation of Folic Acid and *α*-Tocopherol in Lysozyme Particles and Their Bioaccessibility in the Presence of DNA

**DOI:** 10.3390/antiox12030564

**Published:** 2023-02-24

**Authors:** Lingling Ma, Tiecheng Gao, Hao Cheng, Ning Li, Weining Huang, Li Liang

**Affiliations:** 1 State Key Lab. of Food Science and Technology, Jiangnan University, Wuxi 214122, China; 2 School of Food Science and Technology, Jiangnan University, Wuxi 214122, China; 3 Fujian Zunjin Health Science and Technology Co., Ltd., and IBF International Inc., Quanzhou 362200, China

**Keywords:** lysozyme, folic acid, *α*-tocopherol, DNA coating, encapsulation, bioaccessibility

## Abstract

Protein particles have been reported as the potential carriers for the co-encapsulation of bioactive components. In this study, lysozyme, a basic protein, was used to simultaneously encapsulate folic acid and *α*-tocopherol at pH 4.0. The encapsulation efficiency and loading capacity of folic acid or *α*-tocopherol increased with its respective concentration. Folic acid had no influence on the encapsulation of *α*-tocopherol. However, the encapsulation of folic acid was improved by *α*-tocopherol below 40 μg/mL but reduced by *α*-tocopherol at higher concentrations. The encapsulation by lysozyme shielded folic acid, *α*-tocopherol, or both partially from the attack of 2,2′-azino-bis-3-ethylbenzthiazoline-6-sulphonic acid (ABTS) radical cation. No masking effect of lysozyme encapsulation on *α*-tocopherol was found in DPPH antioxidant activity assay. Furthermore, the DNA coating was used to improve the dispersion of lysozyme with folic acid and *α*-tocopherol. The lysozyme/DNA particles with folic acid and *α*-tocopherol showed a homogenous size distribution of 180–220 nm with ζ-potential values between −33 and −36 mV. The release and bioaccessibility of folic acid in lysozyme/DNA with *α*-tocopherol were similar to that of folic acid alone, while the release of *α*-tocopherol was delayed and its bioaccessibility was improved by encapsulation in lysozyme/DNA with folic acid. The data gathered here would provide guidance for the use of lysozyme-based co-encapsulating carriers in the development of functional foods.

## 1. Introduction

Bioactive components (BCs) have health benefits and can be used to prevent or delay the onset of chronic diseases. Encapsulation in a carrier has been widely used for the protection and delivery of a single BC. When more than one BCs are co-encapsulated in a complex carrier, multiple health benefits, synergistic bioactivity and improved stability have been reported. For example, oil-water-oil emulsions have the inner aqueous phase and the oil phase for respective encapsulation of hydrophobic and hydrophilic vitamins [1]. Recently, protein-based homogenous particles were used for the co-encapsulation of bioactive components, including *α*-tocopherol/resveratrol in zein particles as well as *α*-tocopherol/naringenin in whey protein particles [2,3].

Lysozyme is a stable basic protein with 129 amino acid residues and an isoelectric point around pH 10.7. About 90.8% vitamin D_3_ was encapsulated in lysozyme complex particles with β-lactoglobulin, a major whey protein in bovine [4]. When encapsulated in lysozyme/κ-carrageenan particles, the release of curcumin in simulated intestinal fluid was much greater than that in simulated gastric fluid [5]. The reports focus on the encapsulation and delivery of a single BC in lysozyme-based particles [6,7]. To our knowledge, the use of lysozyme for the co-encapsulation of BCs is rare. The complexation of DNA with proteins exists in nature and is also important for nanotechnology. DNA-coated zein particles have been used for the encapsulation and protection of kaempferol or α-tocopherol [8]. Lysozyme-induced charge neutralization of DNA led to the formation of compact particles in a globule shape [9]. It is thus possible to develop the potential carriers for the delivery of BCs using lysozyme and DNA complex particles.

Folic acid is a member of the vitamin B family and can effectively prevent neural tube defects and megaloblastic anemia [10]. It has been reported that the bioavailability of folic acid was 50% in food, while the bioavailability of folic acid supplement was 85% when mixed with diet [11]. The high entrapment of folic acid was obtained using hetero protein particles of β-lactoglobulin and lactoferrin at pH 5.5 [12]. *α*-Tocopherol is a vitamin E with antioxidant, immunomodulatory and anti-inflammatory activity, but its application is limited due to hydrophobicity and low bioavailability [13]. The binding of *α*-tocopherol with lysozyme was synergistically driven by enthalpy and entropy [14]. The co-encapsulation with naringenin in WPI particles improved the stability and bioaccessibility of *α*-tocopherol [3].

In this study, folic acid and *α*-tocopherol as the models of bioactive components were co-encapsulated using lysozyme. Antioxidant activity was analyzed by using 2,2′-azino-bis-3-ethylbenzthiazoline-6-sulphonic acid (ABTS) and 2,2-diphenyl-1-picrylhydrazyl (DPPH) radical scavenging capacity. Physical stability of lysozyme particles with folic acid and *α*-tocopherol and bioaccessibility of two bioactive compounds were improved by addition of DNA. The data gathered here should be useful for the co-delivery of bioactive components based on biopolymer-based carriers.

## 2. Materials and Methods

### 2.1. Materials

Lysozyme (Biotechnology grade), folic acid (≥99%) and DNase I (2000 U/mg) were purchased from MASKLIN Co. (Shanghai, China). Salmon sperm DNA, DPPH, pepsin (500 U/mg, from porcine stomach mucosa) and pancreatin (from porcine pancreas, 4 × USP specifications) were procured from Sigma-Aldrich Co. (Shanghai, China). *α*-Tocopherol and ABTS were purchased from Sangon Bioengineering Co., Ltd. and Aladdin Bio-Chem Technology Co., Ltd. (Shanghai, China), respectively. Other reagents were analytical grade and obtained from SinoPharm CNCM Ltd. (Shanghai, China).

### 2.2. Sample Preparation

Lysozyme at 1% (*w*/*v*) was prepared by dispersing protein powder into ultrapure water and adjusted the pH to 4.0 under stirring. Stock solution of folic acid at 120 µg/mL was dissolved in ultrapure water at pH 7.0. Stock solution of *α*-tocopherol at 4 mg/mL was dissolved in ethanol. Lysozyme-folic acid or lysozyme-*α*-tocopherol particles were prepared according to the previous methods [15]. Precisely, adding the stock solution of folic acid or *α*-tocopherol into diluted lysozyme solution, adjusting the pH to 4.0 and stirring for 1 h. The stock solutions of folic acid and *α*-tocopherol were sequentially added into the lysozyme solution at 1 h intervals to obtain a lysozyme-folic acid-*α*-tocopherol ternary system. The final concentrations of lysozyme, folic acid and *α*-tocopherol were 0.2%, 20–60 µg/mL and 30–80 µg/mL, respectively.

Preparation of lysozyme-DNA particles with folic acid or *α*-tocopherol referenced the previous methods [8]. DNA was dissolved in 0.01 M NaOH at 0.15%, and adjusted the pH to 4.0. Adding lysozyme particles into 0.15% DNA solution adjusted the pH to 4.0 after stirring for 1 h.

### 2.3. Encapsulation Efficiency of Folic Acid and α-Tocopherol

Folic acid and *α*-tocopherol were quantified by measuring the absorbance at 350 and 284 nm, respectively, on a UV1800 UV–Vis spectrophotometer (Shimadzu Corporation, Tokyo, Japan). The encapsulation efficiency of folic acid in lysozyme particles was estimated by adopting the previous methods [3,16]. Precisely, the lysozyme/folic acid/*α*-tocopherol samples were centrifuged at 100,000× *g* at 4 °C for 30 min, and the supernatants were collected. Folic acid in the supernatants was measured by absorbance at 350 nm. *α*-Tocopherol was extracted from the supernatants by mixture with hexane at a volume ratio of 1:2 under votexing for 2 min followed by centrifugation at 3500× *g* for 5 min, and 1 mL of the supernatant was dried at nitrogen atmosphere and dissolved in 1 mL methanol for the absorbance measurement at 284 nm. Encapsulation efficiency of folic acid and *α*-tocopherol were calculated as follows,

(1) $$\mathrm{Encapsulation}\;\mathrm{efficiency}\;(\%)=\frac{Free\;folic\;acid\;or\;\alpha \text{-}tocopherol}{Folic\;acid\;or\;\alpha \text{-}tocopherol\;in\;itial\;input}\times 100$$

(2) $$\mathrm{Loading}\;\mathrm{capacity}\;(\mathrm{mg}/g)=\frac{Folic\;acid/\alpha \text{-}tocopherol\;encapsulated\;in\;particles\;(mg)}{Weight\;of\;lysozyme\;(g)}$$

### 2.4. Particle Characterization

Size distribution by intensity and ζ-potential of particles was analyzed at 25 °C on a NanoBrook Omini analyzer (Brookhaven Instrument, New York, NY, USA) at a scattering angle of 173°. Phase analysis light scattering was employed to ζ-potential.

Samples were diluted and dropped on silicon wafer and air-dried at room temperature. The microstructure of different composite particles was observed by atomic force microscopy (AFM).

### 2.5. Antioxidant Activity

Antioxidant activity was measured by ABTS and DPPH assays [17]. ABTS·^+^ was produced by mixing 7.4 mM ABTS and 2.6 mM K_2_S_2_O_8_ in the dark for 12 h and diluted by 50 times with 10 mM phosphate buffer at pH 7.4. After samples were mixed with ABTS·^+^ solution at a volume ratio of 1:10 for 6 min, the absorbance at 729 nm was measured. DPPH at 80 μM was prepared by dissolving in ethanol. After DPPH solution was added to samples at a volume ratio of 4:3 and allowed to react for 60 min in the dark, the absorbance was recorded at 517 nm. Scavenging activity was calculated by using the formula,

(3) $$\mathrm{Scavenging}\;\mathrm{capacity}\;(\%)=\frac{Ac-As}{Ac}\times 100$$

where Ac and As are the absorbances of the radical solution without and with the sample, respectively. The ABTS·^+^ and DPPH· scavenging capacities were expressed as Trolox equivalents using Trolox as a standard. The ABTS·+ scavenging capacity calibration curve of the Trolox is y = 0.0072x + 0.0087 (R^2^ = 0.9924), and the DPPH· scavenging capacity calibration curve of the Trolox is y = 0.0115x − 0.0052 (R^2^ = 0.9954).

### 2.6. In Vitro Release

Release of folic acid and *α*-tocopherol from lysozyme and lysozyme-DNA particles was carried out with dialysis bags (14 kDa MWCO, Sinopharm Chemical Reagent Co., Ltd., Shanghai, China) according to the diffusion method [18]. Simulated gastric fluid (SGF) containing 0.2% NaCl and 3.2 mg/mL pepsin at pH 2 and simulated intestinal fluid (SIF) containing 3.2 mg/mL pancreatin 150 U/mL DNase I and 0.15 mM MgSO_4_ in 20 mM potassium phosphate buffer at pH 7 were formulated [19,20]. Ethanol was added to the medium at a volume ratio of 7:1 to obtain a dissociation condition for *α*-tocopherol in SGF and SIF. Exactly 7.5 mL of samples were mixed with 7.5 mL of pre-heated SGF in 37 °C, and the mixture at pH 2 was digested at 150 rpm for 2 h. Then 1 M NaOH solution was used to adjust the pH to 7.0, and 15 mL of SIF was added and digested for 4 h. At regular intervals, 1 mL of dissolution medium was collected, and the same amount of fresh medium was added to maintain the volume. The release of folic acid or *α*-tocopherol in the sink medium was calculated using the following equation:

(4) $$\mathrm{Release}\;(\%)=\frac{Content\;of\;folic\;acid\;or\;\alpha \text{-}tocopherol\;in\;dissolution\;medium}{Initial\;amount\;of\;folic\;acid\;and\alpha \text{-}tocopherol\;inside\;dialysis}\times 100$$

### 2.7. Bioaccessibility

The bioaccessibility of folic acid and *α*-tocopherol was determined by adopting the method of Khan et al. [18]. Exactly 5 mL of free folic acid, *α*-tocopherol and folic acid/*α*-tocopherol-loaded particles was added to 5 mL of SGF and incubated for 2 h at 37 °C, then the pH of dispersion was adjusted to 7.0 with 1 M NaOH and 10 mL of SIF was added and incubated for 4 h. The digesta was centrifuged at 10 °C and 10,000× *g* for 30 min. Folic acid and *α*-tocopherol in the supernatant were measured using the method described in Section 2.3. Their bioaccessibility was calculated by the following equation:

(5) $$\mathrm{Bioaccessibility}\;(\%)=\frac{Content\;of\;folic\;acid\;and\;\alpha \text{-}tocopherol\;in\;supernatant}{Initial\;content\;of\;folic\;acid\;and\;\alpha \text{-}tocopherol\;in\;the\;samples}\times 100$$

### 2.8. Statistical Analysis

Statistical analysis was performed using SPSS (IBM Co., Ltd., New York, NY, USA).

## 3. Results and Discussion

### 3.1. Encapsulation of Folic Acid and α-Tocopherol by Lysozyme

**Figure 1:**
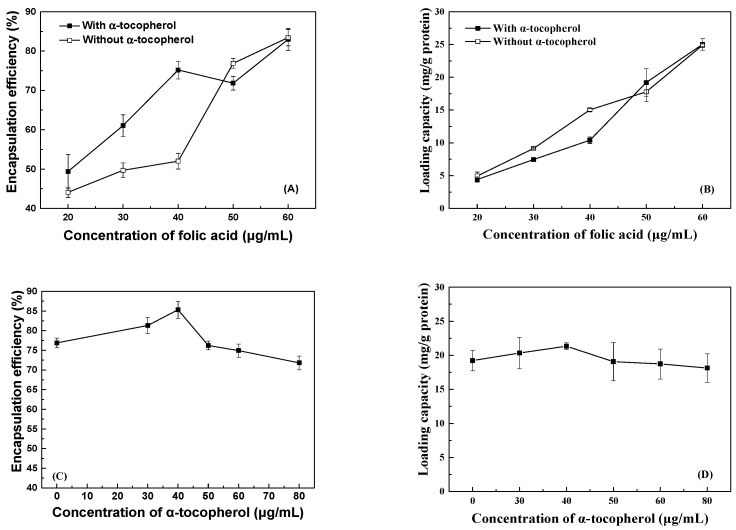
Encapsulation efficiency and loading capacity of folic acid at various concentrations in lysozyme particles without and with 80 μg/mL *α*-tocopherol (**A**,**B**) and of 50 μg/mL folic acid in lysozyme particles with various concentrations of *α*-tocopherol (**C**,**D**).

The encapsulation efficiency of folic acid increased gradually from 44% to 83% (Figure 1A) and the loading capacity of folic acid increased from 4 to 25 mg/g (Figure 1B) as its concentration increased from 20 to 60 μg/mL. The encapsulation efficiencies in this study were higher than those reported in an earlier study; the encapsulation efficiency of folic acid at 20 mg/mL was found to be 21.6% by Mohammed et al. using folic acid-sporopollenin microcapsules prepared via the vacuum technique [21]. When the concentrations of folic acid were between 20 and 40 μg/mL, the increase in the folic acid encapsulation efficiency was more pronounced and the encapsulation efficiencies of folic acid were greater in the presence than absence of 80 μg/mL *α*-tocopherol (Figure 1A). However, the effect of *α*-tocopherol was contrary when the concentration of folic acid was 50 μg/mL. The encapsulation efficiencies of folic acid at 60 μg/mL were similar in the absence and presence of *α*-tocopherol. The effect of *α*-tocopherol on the loading capacity of folic acid (Figure 1B) was less pronounced than that on the encapsulation efficiency (Figure 1A). When the concentration of folic acid was fixed at 50 μg/mL, its encapsulation efficiency increased to 85% as the concentration of *α*-tocopherol increased to 40 μg/mL and then decreased gradually to 71% as the *α*-tocopherol concentration increased to 80 μg/mL (Figure 1C). In addition, the loading capacity of folic acid remained at 19 mg/g in the absence and presence of *α*-tocopherol at various concentrations (Figure 1D).

**Figure 2:**
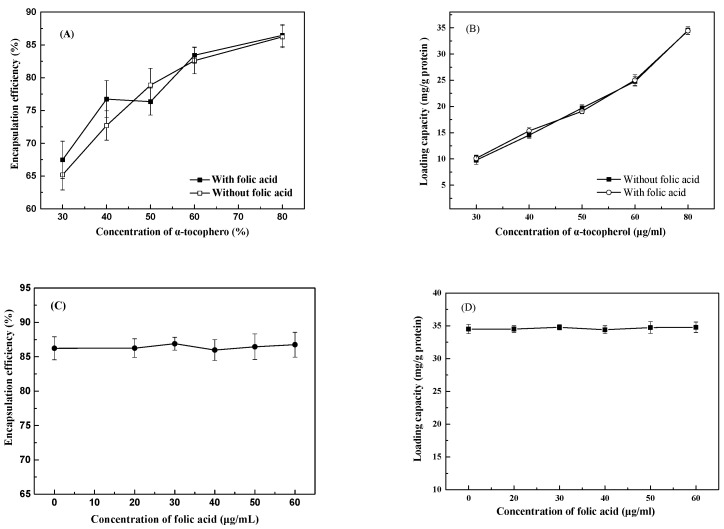
Encapsulation efficiency and loading capacity of *α*-tocopherol at various concentrations by lysozyme without and with 50 μg/mL folic acid (**A**,**B**) and of 80 μg/mL *α*-tocopherol by lysozyme with various concentrations of folic acid (**C**,**D**).

The encapsulation efficiency of *α*-tocopherol increased from 65% to 86% (Figure 2A) and the loading capacity of *α*-tocopherol increased from 10% to 34% (Figure 2B) as its concentration increased from 30 to 80 μg/mL, which was independent of the presence of 50 μg/mL folic acid. These results suggest that the encapsulation of *α*-tocopherol by lysozyme was not saturated because the molar concentration of lysozyme is much greater than that of *α*-tocopherol. The encapsulation efficiencies obtained in this study were much higher than those of about 42–47% reported in an earlier study of 200 μM *α*-tocopherol loaded in WPI nanoparticles at protein concentration of 0.05–1% and pH 7 [2]. Additionally, the encapsulation efficiency and loading capacity of 80 μg/mL *α*-tocopherol were not affected by folic acid at 20–60 μg/mL (Figure 2C,D). These results suggest that folic acid had no influence on the encapsulation of *α*-tocopherol by lysozyme.

### 3.2. Antioxidant Activity of Lysozyme and Folic Acid/α-Tocopherol

#### 3.2.1. ABTS Assay

**Figure 3:**
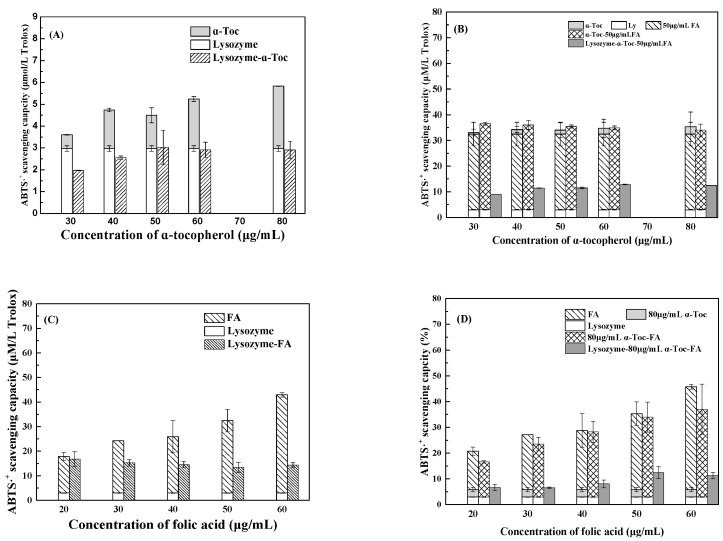
ABTS·^+^ scavenging capacity of 0.2% lysozyme, 30–80 μg/mL *α*-tocopherol (*α*-Toc), 20–60 μg/mL folic acid (FA) and their binary (**A**,**C**) and tertiary mixtures (**B**,**D**).

Figure 3A shows that lysozyme at 0.2% exhibited 2.97 μmol/L Trolox ABTS·^+^ scavenging capacity. The scavenging capacity of *α*-tocopherol increased from 0.63 to 2.86 μmol/L Trolox as its concentration increased from 30 to 80 μg/mL. When the concentrations of *α*-tocopherol were between 50–80 μg/mL, the scavenging capacities of lysozyme-*α*-tocopherol mixtures were similar to those of lysozyme alone, because the encapsulation by lysozyme shielded *α*-tocopherol from the attack of ABTS·^+^ in the aqueous solution. The masking effect was previously reported for the encapsulation of *α*-tocopherol by zein and *N*-vinylpyrrolidone/triethylene glycol dimethacrylate [2,22]. When the concentrations of *α*-tocopherol were 30 and 40 μg/mL, the scavenging capacities of lysozyme-*α*-tocopherol mixtures were less than their individual sum, and their difference was greater than the scavenging capacity of *α*-tocopherol. These results suggest that the encapsulation of *α*-tocopherol at low concentrations may cause the aggregation of lysozyme and thus shield the protein groups from the attack of ABTS·^+^ in the aqueous solution.

Figure 3C shows that ABTS·^+^ scavenging capacity of folic acid increased from 14.89 to 39.96 μmol/L Trolox as the folic acid concentration increased from 20 to 60 μg/mL. When the concentration of folic acid was 20 μg/mL, the scavenging capacities of lysozyme-folic acid mixtures were similar to their individual sum, suggesting an additive effect. At higher concentrations of folic acid, the scavenging capacities of lysozyme-folic acid mixtures were less than their individual sum, but the difference was less than those of folic acid alone. These results suggest that the encapsulation by lysozyme partially shield folic acid from the attack of ABTS·^+^ in the aqueous solution. In a study on the co-encapsulation of folic acid with vitamin C, their antioxidant activity was found to be increased by coating with chitosan [23].

As shown in Figure 3B, when the concentrations of folic acid and *α*-tocopherol were 50 and 30 μg/mL, respectively, ABTS·^+^ scavenging capacities of their mixtures were greater than their individual sum, suggesting a synergistic effect. At higher concentrations of *α*-tocopherol, ABTS·^+^ scavenging capacities of their mixtures were similar to their individual sum, suggesting an additive effect [24]. The additive effect was also observed for 80 μg/mL *α*-tocopherol and 30–50 μg/mL folic acid (Figure 3D). However, when the concentrations of folic acid were 20 and 60 μg/mL, the scavenging capacities of their mixtures were less than their individual sum, suggesting an antagonistic effect [17]. ABTS·^+^ scavenging capacities of lysozyme-*α*-tocopherol-folic acid mixtures were significantly less than the sum of lysozyme and *α*-tocopherol-folic acid mixtures at various concentrations of *α*-tocopherol or folic acid (Figure 3C,D), suggesting the encapsulation by lysozyme shielded the vitamins mostly from the attack of ABTS·^+^ in the aqueous solution.

#### 3.2.2. DPPH Assay

**Figure 4:**
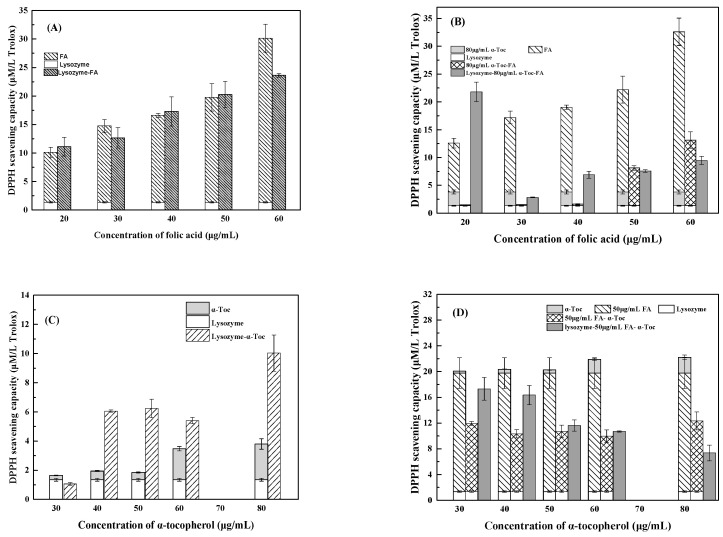
DPPH scavenging capacity of 0.2% lysozyme, 30–80 μg/mL *α*-tocopherol (*α*-Toc), 20–60 μg/mL folic acid (FA) and their binary (**A**,**C**) and tertiary mixtures (**B**,**D**).

The DPPH radical scavenging capacity of 0.2% lysozyme was 1.35 µmol/L Trolox (Figure 4A). The scavenging capacity of folic acid increased from 8.79 to 28.78 µmol/L Trolox as its concentration increased from 20 to 60 μg/mL. When folic acid concentrations were between 20 and 50 μg/mL, the scavenging capacity of lysozyme and folic acid mixture was similar to the sum of their individuals, showing an additive effect. At 60 μg/mL folic acid, the scavenging capacity of lysozyme-folic acid mixture was lower than the sum of the two, suggesting the masking effect of lysozyme on folic acid. Figure 4C shows that the DPPH· scavenging capacity of α-tocopherol increased from 0.3 to 2.45 µmol/L Trolox as its concentrations increased from 30 to 80 µmol/L. The masking effect was observed for α-tocopherol at 30 μg/mL by lysozyme mixture. At higher concentrations of α-tocopherol, the scavenging capacities of lysozyme-α-tocopherol mixtures were higher than their sum, suggesting synergistic antioxidant capacity. Similarly, resveratrol and zein had synergistic antioxidant capacity in the determination of DPPH· scavenging assay, while hollow zein shielded the resveratrol mostly from the attack of ABTS·^+^ [18]. As shown in Figure 4B,D, the DPPH· scavenging capacities of folic acid-α-tocopherol mixture were less than their sum, suggesting an antagonistic effect [25]. At different concentrations of α-tocopherol or folic acid, the masking effect was also observed for lysozyme-α-tocopherol-folic acid mixture (Figure 4B,D), except for the synergistic effect at 20 µg/mL folic acid and 80 µg/mLα-tocopherol (Figure 4B).

### 3.3. Lysozyme-DNA Complex Particles

**Figure 5:**
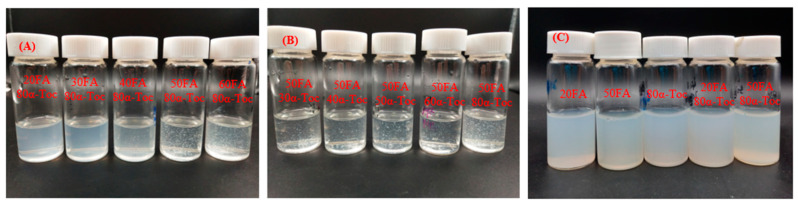
Visual observation of lysozyme with 80 μg/mL *α*-tocopherol (*α*-Toc) and various concentrations of folic acid (**A**); lysozyme with 50 μg/mL folic acid (FA) and various concentrations of *α*-tocopherol (**B**); lysozyme and DNA with 20 and 50 μg/mL folic acid, 80 μg/mL *α*-tocopherol and their mixture (**C**); and after storage at 25 °C for 2 days. The concentration of DNA is 0.075%.

**Figure 6:**
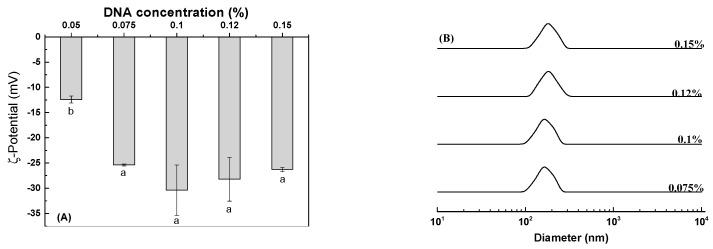
ζ-Potential (**A**) and size distribution (**B**) of lysozyme-DNA particles at various concentrations of DNA. Different letters mean significant differences at *p* < 0.05 in (**A**).

**Figure 7:**
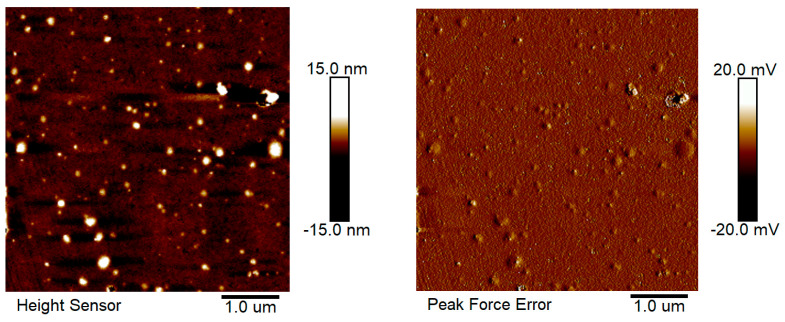
AFM images of lysozyme-DNA particles with the DNA concentration of 0.075%.

As shown in Figure 5A,B, precipitation appeared for lysozyme with folic acid and *α*-tocopherol after storage for 2 days. The dispersions were stabilized upon addition of 0.075% DNA (Figure 5C). Forming protein–DNA structures in solution is a complex dynamic process involving noncovalent forces including electrostatic attraction and hydrophobic interaction. The electrostatic attraction can occur between positively-charged lysozyme and negatively-charged DNA, which is instrumental in controlling the morphology of the formed assemblies [26,27,28]. As the concentration of DNA was 0.05%, the ζ-potential of lysozyme and DNA particles was −12 mV (Figure 6A). The complex particles had ζ-potential values around −28 mV (Figure 6A) with homogeneous size distribution around 170–191 nm and the PDI value less than 0.2 (Figure 6B, Table S1) when the concentrations of DNA ranged from 0.075% to 0.15%. These results suggest that DNA was distributed in the outer layer of particles and stabilized them in the aqueous solution. The complex particles of lysozyme/κ-carrageenan increased in size as their ratios increased [29]. The sizes of xanthan gum/lysozyme particles increased from 61.7 to 108 nm as their ratios changed from 3:1 to 1:3 [30]. The independence of lysozyme/DNA particle size on DNA concentration (Figure 6B) indicates that DNA at 0.075% is enough to stabilize lysozyme particles in aqueous solution. The lysozyme-DNA particles were regular spherical (Figure 7). Therefore, the concentration of DNA at 0.075% was used for subsequent experiments.

**Figure 8:**
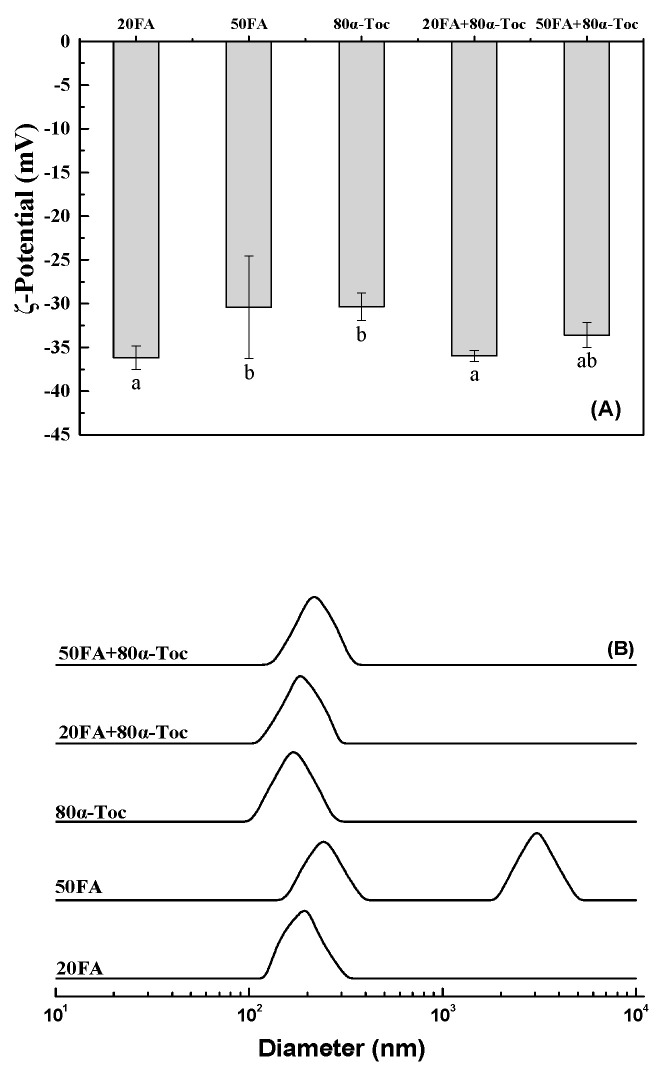
ζ-Potential (**A**) and size distribution (**B**) of lysozyme-DNA particles with 80 μg/mL *α*-tocopherol (*α*-Toc) and/or 20 and 50 μg/mL folic acid (FA). The concentration of DNA is 0.075%. Different letters mean significant differences at *p* < 0.05 in (**A**).

Particle size and surface charge were basic parameters of delivery system [31]. As shown in Figure 8A, lysozyme/DNA particles with 80 μg/mL *α*-tocopherol plus 20 or 50 μg/mL folic acid had ζ-potential values between −33 and −36 mV. The ζ-potential decreases as the folic acid concentration increases. Lysozyme/DNA particles had a size distribution around 168.21 nm with 80 μg/mL *α*-tocopherol, and a size distribution around 168.16 nm with 20 μg/mL folic acid (Table 1). The size of lysozyme/DNA particles gradually increased as the concentration of folic acid increased, with the peaks around 240 and 3090 nm at 50 μg/mL folic acid (Figure 8B). The effect of folic acid on lysozyme/DNA particles is consistent with that of folic acid on *β*-lactoglobulin micro/nanocarriers in the acidic environment; the addition of folic acid increases the particles’ size [32]. Lysozyme/DNA particles had a size distribution around 197.8 or 233.14 nm with 80 μg/mL *α*-tocopherol plus 20 or 50 μg/mL folic acid (Table 1), respectively. These results suggest that the co-encapsulation of folic acid and *α*-tocopherol has the advantage of keeping the homogenous distribution of lysozyme/DNA particles. The effect may be the reason for the inversion impact of *α*-tocopherol on the encapsulation efficiency of folic acid by lysozyme (Figure 1A).

### 3.4. In Vitro Digestion

**Figure 9:**
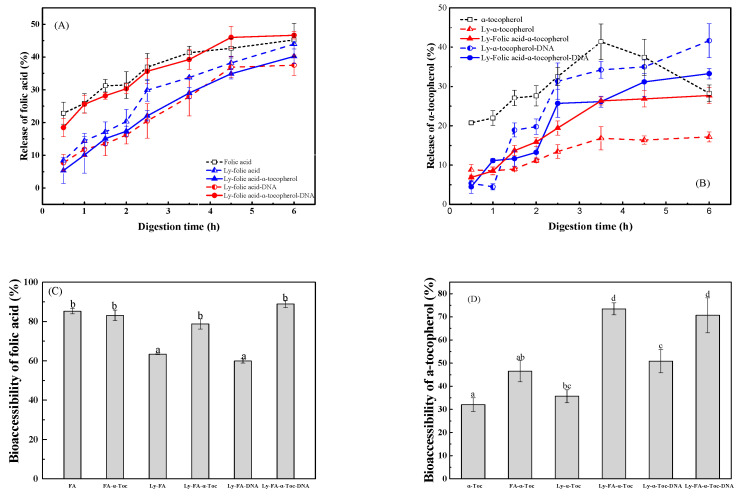
In vitro release and bioaccessibility of folic acid (**A**,**C**)/*α*-tocopherol (**B**,**D**) encapsulated in lysozyme and lysozyme-DNA without or with 80 µg/mL *α*-tocopherol/20 µg/mL folic acid. Different letters mean significant differences at *p* < 0.05 in (**C**,**D**).

The released folic acid increased gradually during GI digestion, being 30% in SGF after 2 h and 45% in SIF after 6 h (Figure 9A). The release was similar for folic acid control and encapsulated in lysozyme-DNA particles with *α*-tocopherol. In comparison, the release of folic acid was slower in the particles of lysozyme without and with *α*-tocopherol and in lysozyme-DNA without *α*-tocopherol. In the particles of lysozyme-DNA, the incorporation of *α*-tocopherol improved the release of folic acid during digestion. However, the release of folic acid (Figure 9A) is significantly less than that (~84%) of folic acid encapsulated in starch/*β*-cyclodextrin microcapsules using spray-drying after 5-min mouth, 60-min gastric condition and 4-h intestinal condition [33]. As shown in Figure 9B, the released *α*-tocopherol reached 28% in SGF after 2 h and 32% in SIF after 2.5 h but then decreased to 28% in SIF after 6 h. This may be due to the interaction between alpha-tocopherol and pancreatic enzymes [34]. The release of *α*-tocopherol was delayed by encapsulation in lysozyme and lysozyme-DNA particles without and with folic acid in SGF. The released *α*-tocopherol from lysozyme particles without and with folic acid were about 17% and 26%, respectively, in SIF after 6 h. The released *α*-tocopherol from lysozyme-DNA particles without and with folic acid increased up to 33% and 42% in SIF after 6 h.

The bioaccessibility of folic acid was 85%, which was not affected by *α*-tocopherol and co-encapsulation with *α*-tocopherol in lysozyme and lysozyme-DNA particles (Figure 9C). However, its bioaccessibility was less upon encapsulation of folic acid alone in lysozyme and lysozyme-DNA particles. These results suggest that the co-encapsulation of *α*-tocopherol could keep the bioaccessibility of folic acid in lysozyme and lysozyme-DNA particles. In the case of *α*-tocopherol, its bioaccessibility was 32%, which was not affected by encapsulation in lysozyme particles (Figure 9D). However, the bioaccessibility of *α*-tocopherol increased to about 48% in the presence of folic acid or by encapsulation in lysozyme-DNA particles. Yin et al. reported that the presence of naringenin improved the bioaccessibility of *α*-tocopherol in whey protein particles [3]. Its bioaccessibility further increased to about 72% by co-encapsulation with folic acid in lysozyme and lysozyme-DNA particles (Figure 9D). This might be because encapsulation in nanoparticles inhibited the precipitation of *α*-tocopherol in SIF and improved its bioaccessibility [35].

## 4. Conclusions

Folic acid and *α*-tocopherol could be co-encapsulated by lysozyme at pH 4. The encapsulation of *α*-tocopherol was not affected by the presence of folic acid, while the low concentrations of *α*-tocopherol improved the encapsulation of folic acid. The highest encapsulation efficiency and loading capacity of folic acid and *α*-tocopherol were 83% and 2.5 mg/g protein and 88% and 3.5 mg/g protein, respectively. The encapsulation by lysozyme shielded *α*-tocopherol and/or folic acid from the attack of ABTS·^+^ to some extent. However, the encapsulation by lysozyme enhanced DPPH radical scavenging ability of tocopherol. The aqueous dispersion of lysozyme with folic acid and *α*-tocopherol was stabilized by coating with DNA. The lysozyme/DNA particles with folic acid and *α*-tocopherol showed a homogenous size distribution of 180–220 nm. The bioaccessibility of folic acid in lysozyme/DNA with *α*-tocopherol was similar to that of folic acid alone, while the bioaccessibility of *α*-tocopherol was improved by encapsulation in lysozyme/DNA particles and the presence of folic acid. The data gathered here would provide guidance for the use of lysozyme-based co-encapsulating carriers in the development of functional foods.

## Figures and Tables

**Table 1:** Mean diameter and PDI of the particles with 0.2% lysozyme and 0.075% DNA in the presence of 80 μg/mL *α*-tocopherol and/or 20 and 50 μg/mL folic acid.

Folic Acid (μg/mL)	α-Tocopherol (μg/mL)	Mean Diameter (nm)	PDI
20	0	168.16 ± 5.10 ^a^	0.19 ± 0.020 ^ab^
50	0	2196.51 ± 138.48 ^d^	0.34 ± 0.02 ^c^
0	80	168.21 ± 5.92 ^ab^	0.18 ± 0.01 ^a^
20	80	197.80 ± 4.12 ^b^	0.17 ± 0.04 ^a^
50	80	233.14 ± 0.32 ^c^	0.20 ± 0.02 ^b^

Different letters mean significant differences at *p* < 0.05 at same column.

## Data Availability

The data presented in this study are available in the article and Supplementary Materials.

## Supplementary Materials

The following supporting information can be downloaded at: https://www.mdpi.com/article/10.3390/antiox12030564/s1, Table S1: Mean diameter and PDI of lysozyme-DNA particles at various concentrations of DNA.
