# Supplementary material for: Encapsulation of Folic Acid and α-Tocopherol in Lysozyme Particles and Their Bioaccessibility in the Presence of DNA
Source: Antioxidants (Basel). 2023 Feb 24;12(3):564. doi: 10.3390/antiox12030564 (PMC10045426; doi:10.3390/antiox12030564)
Supplement: Supplementary file 1 [file antioxidants-12-00564-s001.zip › antioxidants-2192467-supplementary.pdf]

Supplemental data

Table S1. Mean diameter and PDI of lysozyme-DNA particles at various concentrations of DNA.

DNA concentration (%)	Mean diameter（nm）	PDI
0.15	170.24±22.06[a]	0.148±0.024 [a]
0.12	174.84±15.77 [a]	0.138±0.019 [a]
0.1	179.79±9.29 [a]	0.151±0.008 [a]
0.075	191.16±4.55 [a]	0.149±0.004 [a]

Different letters mean significant differences at $p < 0.05$ at same column.
